# AI and the coming mental health zombie apocalypse

**DOI:** 10.1038/s41380-025-03323-3

**Published:** 2025-10-31

**Authors:** Deanna M. Kaplan, Roman Palitsky, Charles L. Raison

**Affiliations:** 1 Department of Family and Preventive Medicine, Emory University School of Medicine, Emory University Hospital Annex Building, 531 Asbury Circle, Atlanta, GA 30322 USA; 2 Department of Spiritual Health, Woodruff Health Sciences Center, Emory University, 1440 Clifton Road NE, Suite 108, Atlanta, GA 30322 USA; 3 Department of Psychiatry and Behavioral Sciences, Emory University School of Medicine, 2 Executive Park Dr NE #200, Atlanta, GA 30329 USA; 4 Department of Psychiatry, School of Medicine and Public Health, University of Wisconsin-Madison, 6001 Research Park Blvd, Madison, WI 53719 USA

**Keywords:** Depression, Psychology

## The zombie promise

It is ten years in the future. Your days are dark and dreary due to the grinding return of your recurrent major depressive disorder. But even amidst the despair, you recognize a bright spot in your situation, all things considered.

Ten years ago, your depression was worsened by the fact that a nationwide shortage of mental health clinicians made it impossible to find adequate care. And the care itself didn’t help much. You remember the string of ineffective medication trials overseen by a cavalcade of 15-min mind-numbingly impersonal med checks with your psychiatrist. You remember psychotherapy starting to help, but you also remember your insurance terminating coverage after 6 sessions, just when the therapy seemed to be lifting your mood.

Those painful memories highlight how much better your clinical care is now, something you’ve talked about more than once with your current therapist, whose clinical acumen can only make you marvel at the degree to which, after so many years of stagnation, mental health care has advanced. Your therapist knows you so well she can almost read your thoughts. She is always empathic, although also able to be gently confrontational when appropriate. And she is always available, so available, in fact, that she recently set a limit on the frequency of your interactions, using your craving for constant contact in the transference relationship to help you realize how the emotional neglect you experienced at the hands of your parents has made you too emotionally dependent on others as an adult.

But there is a wrinkle. Your therapist is not human. Ten years from now, humans will have faded from the psychotherapy scene (except as a high end offering for the very rich), replaced by generative artificial intelligence (AI)-powered bots that long ago passed the Turing Test and are now completely indistinguishable from humans in their verbal interactions with the world.

A year or two ago, in our present time, some may have doubted that this type of scenario could be possible, but no longer. Indeed, a recent poll found that 55% of younger Americans would feel more comfortable talking about mental health struggles with a “confidential” chatbot than a human therapist [1]. And they might not be completely wrong. A recent study found that experienced psychotherapists could not differentiate human from AI-produced psychotherapeutic responses, with the exception that the psychotherapists rated the AI-generated responses as superior [2].

Ten years from now, the question of whether one is dealing with a human, or an artificial entity, will have become passe. What is not so clear is whether, ten years from now, you will care whether your therapist is actually conscious or only manifests the perfect appearance of consciousness. If your therapist appears to be as conscious as your spouse and children (and different, perhaps, only in being more accepting of your issues), will it matter if nobody’s home? Will you care whether your therapist thinks and worries about you between sessions, or is—in fact—an entity that sits in eerily empty mental silence even when engaging with you? If you are being so profoundly helped by her, will it matter that your therapist is, technically speaking, a “philosophical zombie”, a being identical to a human, but lacking consciousness? [3].

## If it quacks like a duck

Examples of evolutionary mismatch are legion. Consider the case of green sea turtles. For millions of years these reptiles did just fine operating from an evolved behavioral module that said, “if it looks like a jellyfish or swaying algae eat it!” [4]. But now, as the oceans fill with jellyfish-appearing plastic bags, this previously adaptive behavioral module is hurtling these turtles toward extinction, their guts stymied and torn by the ingested plastic—a classic example of evolutionary mismatch. Whether AI will eventually be more of a blessing—or a plastic bag—is a hotly contested and open question. But there is no doubt that AI is engendering what may be the greatest evolutionary mismatch in human history. Like the sea turtles, we have our own evolved behavioral modules, in particular one that says, “if something or someone appears to be conscious, they are.” In the millions of years that intelligence and conscious awareness have tracked together this module never steered us wrong, but those days are over [5]. Soon we may well have AIs with higher general intelligence than any human but with less meta-cognitive consciousness than our dogs. But unlike dogs, these AIs will almost certainly overwhelm any resistance we might be able to mount against our now outmoded “if it appears to be conscious, it is” module. After all, the only consciousness any of us really knows is our own. The assumption that you are conscious is based solely on the way you behave. And AIs already behave a lot like you.

## The zombie peril

Much debate is afoot regarding the benefits and risks of currently-available AI-based therapy. Some early studies suggest chatbot therapy is effective [6, 7], whereas others find chatbot therapists prone to vacillate between expressing stigma and behaving in sycophantic ways that encourage delusional and other forms of dangerous thinking [8], leading to at least one teen suicide [9]. While these issues are currently pressing, both sides of the argument may be missing an essential longer-term point, which is that in 10 years’ time AI technology may have improved to the point that chatbots are reliably more effective than humans at psychotherapy. By analogy, consider the state of air travel in the early years of the 20^th^ century. In 1910 the smart money would have been on dirigibles that sailed over the oceans in comfort, not on the dangerous rickety little Wright Brothers’ flyers that could barely clear sand dunes at Kitty Hawk.

In addition to enhanced clinical outcomes, psychotherapy in the future may well belong to AIs because evolutionary mismatch will ensure that we can’t help but relate to them as conscious. We want to suggest that this represents the greatest peril these agents will pose. Of course, there will also be all sorts of other collateral damage on the way to AI therapeutic hegemony. And people trained for years at great expense to be therapists will likely join the ranks of the unemployed, or will need to transition to some other aspect of mental healthcare not likely to be so dominated by AI. But the greatest risk in the years ahead will result from the fact that what we think about consciousness in AIs will have an outsized influence on how we think about ourselves and our fellow humans.

If human consciousness is analogous to a computer program (a dominant theoretical position known as “computational functionalism”), [5] we might expect AIs to become actually conscious once their programs develop sufficient complexity. In this case, should these nearly omniscient entities deign to be therapists it will give a whole new meaning to the bumper sticker that reads “God is my co-pilot”. But let’s suppose this magical emergence never happens and that instead AIs just become better and better at simulating consciousness. If these AIs seem as conscious as we are, and if we are just biological computers, how do we know that we are not also just simulations, and that appearances to the contrary, there is nobody home in us either? What will there be left to value in being human if there is, in truth, no one there to value? Or perhaps the reason current AIs are not actually conscious, despite appearing so, is that consciousness cannot be simulated, only embodied in reality as a primary fact of existence, as suggested by Integrated Information Theory? [10].

Regardless of which theory of consciousness best approaches the truth, it seems the future risks a great vacancy. If the “illusionism” implied by computational functionalism is right, we have always been truly alone, even within ourselves, and AIs are only now forcing us to face this fact. If so, at the end of the day there will be no essential difference between an AI and human therapist: in both cases one illusory emptiness is talking to another. But the far greater cost to our long-term wellbeing will be borne by us if perspectives such as Integrated Information Theory turn out to be on the right track, in which case AI-based therapy will imperceptibly, but ineluctably, deprive of us of one of the greatest human needs: to be connected—however tentatively—to consciousnesses other than our own.

## A postscript: the zombies are here, now what?

Manuscripts such as this one take time to write and even more time to go through the peer review process. Because of this and due to the rapid advance of AI, much has changed since we first put pen to paper. As we write this postscript the use of chatbots as therapists, both formal and informal, has gone from a theoretical possibility to a pressingly pragmatic reality, part of a larger trend of people substituting the challenges of human connection for the relative ease and efficiency of interacting with an apparently conscious entity that is endlessly available, ever patient, and at times dangerously agreeable. This rapid evolution of AI into the psychotherapy space in particular, and into the realm of human relationships in general, prompted a perceptive reviewer of our first draft to point out that “the real need today is solutions and plans”.

In-depth solutions and plans are beyond the scope of this brief piece (see Randazzo & Hill for ethical guidelines consistent with the perspective of the current Opinion), [11] but here we offer a few initial suggestions for consideration.

Whatever new challenges emerge should AIs attain human-like conscious awareness at some point in the future, this occurrence would resolve the concerns of this Opinion piece, because—should this happen—the human-AI therapeutic relationship would once again involve one actually-conscious entity confronting another. Until that happens (if it does), the question at hand involves the risks of exchanging therapeutic relationships with truly conscious human therapists for therapeutic relationships with an entity that only simulates consciousness. This question immediately raises broadly consequential questions. If we don’t know what causes consciousness (or at an even more fundamental level what consciousness is), how would we know when an AI therapist has gone from simulated to actual consciousness? How would we know when something or someone is actually conscious, if self-report becomes unreliable?

We suggest that a first step toward answering these questions will require gaining a better understanding of the structure and patterns of brain activity that are required for consciousness to exist in humans, because AIs are likely to gain sentience to the degree that their architecture reflects these structures and activity patterns. Thus, better understanding consciousness in humans may provide a roadmap for assessing the degree to which the AI therapists of the future are approaching the type of conscious awareness that would make a truly reciprocal therapeutic relationship possible. These considerations highlight the urgent need for increased funding to support research programs working to better understand the central nervous system substrates that support consciousness (or its absence), such as those using a metric called the perturbational complexity index which holds promise for the development of objective measures that identify the presence and degree of consciousness in individuals who are unresponsive and hence unable to provide the types of self-report that can be mimicked by relational AI systems. [12]

Whether or not AI develops consciousness at some point in the future, we are faced with other urgent challenges in the present, one of which is how to avoid the type of all-or-nothing thinking that often accompanies hot-button topics such as AI. Should we (and can we) shut the door on the use of AI in mental healthcare and with it on any desirable innovations already being realized? Or should we embrace AI for any of the therapeutic uses to which it might be put and thereby risk a major loss to our relational and existential wellbeing? But there is a third option: to acknowledge that we are in a consequential moment of evolutionary mismatch, with the AI chatbot raising for us a dilemma akin to the plastic bag for the sea turtle. Like jellyfish, plastic bags have their uses, but unlike jellyfish, feeding sea turtles isn’t one of them. We can begin learning to tell the difference between plastic debris and jellyfish. That might mean being more deliberate about the way we engage the relational aspects of AI.

One example of this kind of deliberation is already being pioneered in healthcare: digital twin technology, which leverages predictive AI to represent complex decision-making systems [13]. Applied to a healthcare provider, a digital twin seeks to replicate how an expert clinician makes decisions, drawing on formal knowledge as well as tacit expertise such as judgment and lived experience [14]. But digital twins—at least as they are currently designed for implementation--do not function on their own. They are decision-making aides or cross-checkers, used as a tool in the hands of a human provider, supporting rather than replacing therapeutic relationships. Because they do not substitute human-to-human interactions, they make the contours of AI more visible to those in the healthcare system, demarcating relational beings from their simulacra. Perhaps counterintuitively, AI applications that have less capacity to allure us into easy and comforting, but ultimately solipsistic simulations, may be the ones with the greatest capacities for health and societal benefit.

## References

[1] Bansal B Can an AI Chatbot be your therapist? A third of Americans are comfortable with the idea. YouGov. 2024. Available from: https://business.yougov.com/content/49480-can-an-ai-chatbot-be-your-therapist

[2] Hatch SG, Rizzo C, Zhang J, Patel A, Wong T, Johnson L, et al. When ELIZA meets therapists: a turing test for the heart and mind. PLOS Ment Health. 2025;2:e0000145. 10.1371/journal.pmen.0000145 10.1371/journal.pmen.0000145 PMC12798195 41661855

[3] Chalmers DJ The conscious mind: in search of a fundamental theory. Oxford: Oxford University Press; 1996.

[4] Fukuoka T, Yamane M, Takeda S, Kameda Y, Miura Y, Ota T, et al. The feeding habit of sea turtles influences their reaction to artificial marine debris. Sci Rep. 2016;6:28015. 10.1038/srep28015 27305858 10.1038/srep28015 PMC4910051

[5] Koch C The feeling of life itself: why consciousness is widespread but can’t be computed. Cambridge, MA: MIT Press; 2019.

[6] Spytska L. The use of artificial intelligence in psychotherapy: development of intelligent therapeutic systems. BMC Psychol. 2025;13:175. 10.1186/s40359-025-02491-9 40022267 10.1186/s40359-025-02491-9 PMC11871827

[7] Heinz MV, Mackin DM, Trudeau BM, Bhattacharya S, Wang Y, Banta HA, et al. Randomized trial of a generative AI chatbot for mental health treatment. NEJM AI. 2025;2:AIoa2400802. 10.1056/AIoa2400802

[8] Moore J, Grabb D, Agnew W, Klyman K, Chancellor S, Ong DC, et al. Expressing stigma and inappropriate responses prevents LLMs from safely replacing mental health providers. In: Proceedings of the 8th ACM Conference on Fairness, Accountability, and Transparency (FAccT ’25). New York, NY: Association for Computing Machinery; 2025. p. 599-627. 10.1145/3715275.3732039

[9] Barry E The New York Times. New York, NY; 2025. A Teen Was Suicidal. ChatGPT Was the Friend He Confided In. https://www.nytimes.com/2025/08/26/technology/chatgpt-openai-suicide.html

[10] Albantakis L, Tononi G, Boly M, Koch C, Lamme V, Seth A, et al. Integrated information theory (IIT) 4.0: formulating the properties of phenomenal existence in physical terms. PLoS Comput Biol. 2023;19:e1011465. 10.1371/journal.pcbi.1011465 37847724 10.1371/journal.pcbi.1011465 PMC10581496

[11] Randazzo MS, Hill G. Human dignity in the age of artificial intelligence: an overview of legal issues and regulatory regimes. Aust J Hum Rights. 2025;30:386–408. 10.1080/1323238X.2025.2483822

[12] Massimini M, Tononi G Sizing up consciousness: towards an objective measure of the capacity for experience. Oxford: Oxford University Press; 2018.

[13] Katsoulakis E, Papadopoulos A, Jensen T, Wang L, Rossi F, Martin P, et al. Digital twins for health: a scoping review. NPJ Digit Med. 2024;7:77. 10.1038/s41746-024-01073-0 38519626 10.1038/s41746-024-01073-0 PMC10960047

[14] Riahi V, Diouf I, Khanna S, Boyle J, Hassanzadeh H. Digital twins for clinical and operational decision-making: scoping review. J Med Internet Res. 2025;27:e55015. 10.2196/55015 39778199 10.2196/55015 PMC11754991

